# Hypertension in the High-Cardiovascular-Risk Populations

**DOI:** 10.4061/2011/746369

**Published:** 2012-06-21

**Authors:** Samy I. McFarlane, Girardin Jean-Louis, Ferdinand Zizi, Adam T. Whaley-Connell, Olugbenga Ogedegbe, Amgad N. Makaryus, Ilir Maraj

**Affiliations:** ^1^Division of Endocrinology, State University of New York-Downstate Medical Center, Kings County Hospital Center, 450 Clarkson Avenue, Box 50, Brooklyn, NY 11203, USA; ^2^SUNY Downstate Medical Center, Brooklyn, NY 11203, USA; ^3^University of Missouri-Columbia, Columbia, MO 65212, USA; ^4^New York University, New York, NY 10012, USA; ^5^North Shore University Hospital, Manhasset, NY 11714, USA; ^6^Department of Medicine, SUNY Downstate, Brooklyn, NY 11203, USA

Cardiovascular disease (CVD) is the major killer in adults in the USA and around the world. Latest World Health Organization (WHO) data show than an estimated 17.3 million people died from cardiovascular disease in 2008, representing 30% of all global deaths. Of these 7.3 million were due to coronary heart disease and 6.2 million due to stroke. WHO data estimates that, by 2030, almost 23.6 million people will die from CVD, mainly from heart disease and stroke. CVD is projected to remain the leading causes of death worldwide [1]. In the United States alone cardiovascular disease causes one in three (approximately 800,000) deaths each year. Furthermore, the economic impact of CVD is astounding. For example, the total annual costs resulting from cardiovascular diseases are estimated at $444 billion [2].

Among the various risk factors for CVD that include diabetes/insulin resistance, hypertension, dyslipidemia, central obesity, increased inflammation, and procoagulant state, hypertension is the major cause for increased CVD particularly in the high-risk populations including those with diabetes, minority population, elderly, and stroke victims [3].

Accumulating data indicate that BP control is of paramount importance in decreasing CVD risk. For example, reducing systolic BP by 3–5 mm Hg reduces stroke risk by 2-3% and the decrease in morbidity and mortality resulted from a 10 to 12 mm Hg reduction in systolic blood pressure and a 5 to 6 mm Hg in diastolic pressure using data from multiple clinical trials. The estimated benefit from this degree of blood pressure reduction is a 38% reduction in risk of stroke and a 16% percent reduction in risk of coronary disease [4].

 However, in high-risk individuals, such as people with diabetes, minority population, elderly, and stroke victims as well as those with chronic kidney disease (CKD), BP control is largely suboptimal and those patients would require multiple medications, an average of three, in order to achieve BP goal. A target BP of less than 130/80 mm Hg is currently advocated for hypertensive diabetic patients who are at increased risk for CVD including stroke. There is no specific class of antihypertensives that is currently being advocated to be used as a first-line therapy rather a combination of several drug classes to achieve optimal BP control is what is emphasized in these populations [5]. Hypertension in these high-risk populations is generally characterized by increased salt sensitivity, with volume expansion, systolic hypertension, and orthostatic hypotension as well as insulin resistance and microalbuminuria.

 In this special issue we focus on HTN in the high-CVD-risk populations providing the reader with the cutting edge knowledge in this area with an assortment of research and review papers written by renowned scholars in the field. These papers put an emphasis on hypertension as an underlying cause in development of cardiovascular heart disease including stroke, heart failure, acute coronary syndromes, and CKD. The paper also put an emphasis on blood pressure control and prevention of hypertension in different settings and implementing a community-based approach on managing hypertension. Below you will find a brief outline of each paper published in this special issue.

The authors of *“The age dependent contribution of aortic incident and reflected pressure wave to central blood pressure in african-americans”* determined that aging is associated with increase central aortic systolic pressure and pulse pressure which are in turn predictive of cardiovascular events. Wave reflection which mediates both of these pressures is a component that investigators aimed to decrease with different antihypertensive drugs mainly vasodilators. The authors of this paper concluded that decreasing wave reflection is more efficacious in the younger population.

There is ample evidence linking hypertension and coronary heart disease. Hypertension accelerates the development of atherosclerosis and it can destabilize vascular lesions and precipitate acute coronary events. Therefore it is of paramount importance to achieve and maintain good blood pressure control. The authors of “*Management of hypertension among patients with coronary heart disease*” concluded that in order to achieve a target BP in hypertensive patients with coronary heart disease of <130/80 effective combination therapy is required which includes a b-blocker, ACE inhibitor or ARB and possibly a thiazide diuretic. The goal of therapy is to reduce the morbidity and mortality associated with both hypertension and CHD.

Left ventricular hypertrophy is a maladaptive response of long-standing hypertension and can lead to heart failure and sudden death therefore as per authors of “*Left ventricular hypertrophy: major risk factor in patients with hypertension: update and practical clinical applications,”* controlling arterial pressure, sodium restriction, and weight loss independently contribute to regression of left ventricular hypertrophy which in turn improves diastolic function and coronary flow reserve thus decreasing CVD risk. ACE inhibitors, ARBs, and calcium channel antagonists are important medications that contribute in regression of left ventricular hypertrophy and therefore decrease the CVD risk.

The combination of an ARB (olmesartan) and a calcium channel antagonist (azlenidipine) was compared and discussed in the paper “*Differential effects in cardiovascular markers between high dose angiotensin ii receptor blocker monotherapy and combination therapy of ARB and a calcium channel blocker in hypertension (DEAR trial)*.” Based on this study the combination therapy with an ARB and a calcium channel blocker the arterial stiffness assessed by CAVI, LDL-C with a similar reduction in BP. As these markers influence the future risk for cardiovascular events in hypertensive patients the authors concluded that the combination therapy is a reasonable choice for management of hypertension. Hypertension is a known risk factor for development of atherosclerosis which leads to acute coronary syndromes, however, there are risk factors associated with the development of acute coronary syndromes such as genetic risk, insulin resistance, sympathetic hyperactivity and vasoactive substances. The paper *The impact of hypertension on patients with acute coronary syndromes* showed that hypertensive patients with ACS are usually older, females, nonwhite and have high prevalence of comorbidities and as such deserve a tailored approach for management and followup of hypertension.

Different approaches such as healthy lifestyles with appropriate diet and exercise have been shown to reduce the incidence of hypertension in high-risk patients. The paper “*Community based participatory research approaches for hypertension control and prevention in churches”* examined if any of the programs used in healthcare setting could be applied successfully to reduce the incidence and consequences of hypertension in large communities with potentially huge impact on public health. The paper “*Resistant hypertension and obstructive sleep apnea in the primary-care setting”* pointed out that, in African Americans, who are especially a high-risk group for hypertension the prevalence of resistant hypertension is similar to the range of resistant hypertension in the general hypertensive population; however, patients with resistant hypertension were at a greater risk for obstructive sleep apnea as compared to patients with controlled hypertension. The case report of “*A patient with hemoptysis and renal failure”* showed that pulmonary renal syndrome is a direct sequela of hypertensive emergency and that it needs an immediate recognition and treatment. Even in cases when prompt treatment is initiated patients may suffer end-organ damage such as in this case report.

The paper “*Antropometric indices associated with variation in cardiovascular parameters among primary school pupils in Ile-Ife”* showed that age, height, weight, BMI, abdominal circumference correlate significantly with blood pressure with weight being a more viable predictor of SBP and age a more viable predictor of DBP. The paper “*Management of hypertension in high-risk ethnic minority with heart failure*” puts an emphasis on treatment of hypertension on African Americans as a prequel of heart failure, one of the most common causes of morbidity and mortality in the world. As hypertension is an important cause and consequence of chronic kidney disease the paper “*Hypertension in chronic kidney disease: navigating the evidence*” showed that treatment of hypertension in CKD patients should take into consideration the nature of the underlying kidney disease. Patients with diabetic nephropathy or proteinuric nondiabetic kidney disease benefit from treatment with ACE inhibitors or ARBs to a goal blood pressure of <130/80 mm Hg. The authors of this paper concluded that a dual or triple blockade of the RAS should generally be avoided.

In treating hypertension the best approach as per author of “Management of high blood pressure in those without overt cardiovascular* disease utilizing absolute risk scores*” was utilizing absolute risk scores for development of CVD. This approach promises better targeted therapy, simpler more flexible management regiments and superior clinical outcomes in those who do not have an overt disease.

Adding on an extended release nicotinic acid/laropiprant to a statin has shown to reduce significantly both systolic and diastolic BP as opposed to switching to a high-dose rosuvastatin. This was the conclusion reached by the authors of the paper “*Add on statin extended release nicotinic acid/laropiprant but not the switch to high-dose rosuvastatin lowers blood pressure: an open label randomized study.”*

Being that hypertension is a significant health problem affecting African Americans the community outreach programs in general and faith-based programs in particular can be valuable in reaching at-risk groups. The paper HEALS: a faith-based hypertension control and prevention program for African American churches: training of church “*Leaders as program interventionists”* showed that community health educators with adequate training can be an essential partner in community-based hypertension control programs as they may motivate and encourage the individuals to make the behavioral modifications that have an impact on management of hypertension. As we know, cardiovascular disease in general and hypertension in particular are prevalent around the world. The paper “*High prevalence and low awareness of hypertension is a market population in Enugu, Nigeria”* showed that hypertension was prevalent in 42% of the market workers in that particular study and the conclusion was that the high prevalence of hypertension in this group could be explained by their sedentary lifestyle, salt-laden fast food, and obesity. This showed that this population warrants intensive education in hypertension management and the morbidity and mortality associated with it.

The authors of “*Cardiovascular outcomes in patients with normal and abnormal 24 hour ambulatory blood pressure monitoring”* showed that there are significant associations between cerebrovascular events and absent nocturnal dip of less than 10% and high day time DBP, peripheral vascular disease and morning surge of more than 20/15 mm Hg, cardiac arrhythmias and high day time and night time DBP.

Finally, we hope that our readers will enjoy this special issue and benefit from the cutting edge information provided by our astute authors with the bigger goal being enhancing patient care, treatment, and prevention of excess cardiovascular disease related to hypertension in the high-risk populations.

*Samy I. McFarlane* *Samy I. McFarlane* *Girardin Jean-Louis* *Girardin Jean-Louis* *Ferdinand Zizi* *Ferdinand Zizi* *Adam T. Whaley-Connell* *Adam T. Whaley-Connell* *Olugbenga Ogedegbe* *Olugbenga Ogedegbe* *Amgad N. Makaryus* *Amgad N. Makaryus* *Ilir Maraj* *Ilir Maraj*
